# Subcellular plant carbohydrate metabolism under elevated temperature

**DOI:** 10.1093/plphys/kiaf117

**Published:** 2025-04-16

**Authors:** Charlotte Seydel, Martin Heß, Laura Schröder, Andreas Klingl, Thomas Nägele

**Affiliations:** LMU München, Faculty of Biology, Plant Development, Großhaderner Str. 2-4, Planegg 82152, Germany; LMU München, Faculty of Biology, Plant Evolutionary Cell Biology, Großhaderner Str. 2-4, Planegg 82152, Germany; LMU München, Faculty of Biology, Zoology, Großhaderner Str. 2-4, Planegg 82152, Germany; LMU München, Faculty of Biology, Plant Evolutionary Cell Biology, Großhaderner Str. 2-4, Planegg 82152, Germany; LMU München, Faculty of Biology, Plant Development, Großhaderner Str. 2-4, Planegg 82152, Germany; LMU München, Faculty of Biology, Plant Evolutionary Cell Biology, Großhaderner Str. 2-4, Planegg 82152, Germany

## Abstract

In many plant species, exposure to a changing environmental temperature regime induces an acclimation response that ultimately increases thermotolerance. Under elevated temperatures, membrane systems undergo remodeling to counteract destabilizing thermodynamic effects. Elevated temperature also affects photosynthesis and carbohydrate metabolism due to altered protein functions, enzyme activities, and transport across membrane systems. Here, a combination of electrolyte leakage assays and chlorophyll fluorescence measurements was applied to quantify heat tolerance before and after heat acclimation in *Arabidopsis thaliana* under different temperature regimes. Subcellular carbohydrate concentrations were determined through nonaqueous fractionation and 3D reconstruction of mesophyll cells and subcellular compartments using serial block-face scanning electron microscopy. Across temperature regimes between 32 and 38 °C, 7 d of heat acclimation at 34 °C most efficiently increased tissue heat tolerance. Under such conditions, cytosolic sucrose concentrations were stabilized by a shift in sucrose cleavage rates into the vacuolar compartment, while invertase-driven cytosolic sucrose cleavage was efficiently quenched by fructose and glucose acting as competitive and noncompetitive inhibitors, respectively. Finally, this study provides strong evidence for a sucrose concentration gradient from the cytosol to the vacuole, which might directly affect the physiological role and direction of sugar transport across cellular membrane systems.

## Teaser

Leakage assays, chlorophyll fluorescence measurements, and scanning electron microscopy experiments unravel heat-induced regulation of subcellular carbohydrate metabolism.

## Introduction

Changing temperature regimes have diverse effects on plant growth, development, and metabolism. While a sudden and strong temperature drop or increase typically results in irreversible tissue damage and yield loss, a constant and moderate change of temperature induces an acclimation response which increases temperature tolerance. This acclimation response represents a multigenic process and comprises, among others, signaling cascades, reprogramming of photosynthesis, and the primary and secondary metabolism (Herrmann et al. 2019; Garcia-Molina et al. 2020; Seydel et al. 2022). Increasing temperatures due to global warming have been shown to negatively impact the fitness of plants in their natural habitat and, especially in combination with drought, the yield of crop species (Lippmann et al. 2019; Gampe et al. 2021). Thus, understanding and predicting plant heat response and acclimation capacity is a vital component to understanding and dealing with the impact of globally increasing temperatures on plants.

Heat is perceived by a changing membrane permeability which, for example, influences transmembrane calcium flux (Ranty et al. 2016; Sajid et al. 2018). Also, reactive oxygen species, kinase and phosphatase activation, phytohormone cascades, activation of transcription factors, and heat shock proteins (HSPs) are involved in the perception of heat (Iba 2002; Qu et al. 2013). The immediate recognition of a changing temperature regime is central to stabilizing photosynthesis and metabolism. Photosynthesis is known to be a highly temperature-sensitive process, being inhibited both at low and high temperatures (Berry and Björkman 1980). The photosynthetic performance can already be impacted negatively at moderate heat by Rubisco inactivation (Law and Crafts-Brandner 1999; Crafts-Brandner and Salvucci 2000; Salvucci and Crafts-Brandner 2004; Yamori et al. 2014). The thylakoids are influenced by heat-induced changes in membrane properties and the regulatory connection between ATP synthesis and electron transport can be disrupted (Havaux 1996; Pastenes and Horton 1996; Bukhov et al. 1999, 2000). However, actual damage to photosystem II, quantified by measuring the critical temperature at which the minimal chlorophyll fluorescence is rapidly increasing, is mostly occurring between 40 and 55 °C, depending on the plant species and growth environment (Terzaghi et al. 1989; Zhu et al. 2018).

Carbohydrates are direct products of photosynthesis and represent energy sources and substrates for diverse anabolic pathways. Recent work has shown that carbohydrates are also an important factor in the thermomemory of the shoot apical meristem (Olas et al. 2021). Further, it has been shown that RGS1, a plasma membrane located glucose sensor, is connected to the regulation of thermotolerance in tomatoes, and externally applying glucose to the plants actually increased their thermotolerance (Wang et al. 2024). The enormous plasticity of heat responses in carbohydrate metabolism can also be seen when comparing heat treatments of different durations and severity. Comparing moderate with severe transient heat exposure revealed that sucrose phosphate synthase (SPS) activity is negatively associated with the stability of CO_2_ assimilation rates under elevated temperature (Seydel et al. 2022). Another study showed that, in *Arabidopsis*, the amount of primary carbohydrates, e.g. sucrose, raffinose, and maltose, increases after treatment at 40 °C for up to 240 min which represents a shared feature with cold stress (Ahsan et al. 2010). Additionally, within the first 24 h of heat exposure, 15% of upregulated proteins in soybean were found to be related to carbohydrate metabolism, but proteins that were associated with carbon assimilation and photosynthesis were found to be downregulated in the same plants (Ahsan et al. 2010).

Pathways of cellular plant carbohydrate metabolism are located in different subcellular compartments. For example, the Calvin–Benson–Bassham cycle (CBBC) takes place in the chloroplasts, while sucrose biosynthesis is catalyzed in the cytosol. The high degree of compartmentalization of plant cells impacts the analysis and understanding of their metabolic pathways profoundly (Lunn 2007). The method of nonaqueous fractionation (NAF) has been applied to resolve compartment-specific metabolic regulation (Gerhardt and Heldt 1984; Fürtauer et al. 2016; Hernandez et al. 2023). This method allows for continuous quenching of metabolism and prevents enzymatic interconversion of metabolites after sampling and during fractionation. The correlation of metabolite abundance with marker enzyme activity, or marker protein abundance, provides information about relative metabolite distributions over analyzed compartments (Fürtauer et al. 2019). If available, absolute amounts of metabolites can then be multiplied with relative distributions to provide an estimate of compartment-specific absolute metabolite amounts. A current limitation is the estimation of effective subcellular metabolite concentrations which also needs to consider compartment-specific volumes.

In the present study, we have combined leakage assays and chlorophyll fluorescence measurements with the NAF methodology and serial block-face scanning electron microscopy (SBF-SEM) to quantify thermotolerance and photosynthetic efficiency together with carbohydrate concentrations in the plastids, cytosol, and vacuole of leaf mesophyll cells of *Arabidopsis thaliana*. Effective compartment-specific concentrations were determined before and after acclimation to 34 °C to unravel heat-induced regulation of subcellular carbohydrate metabolism.

## Results

### Electrolyte leakage indicates efficient Arabidopsis heat acclimation between 32 and 34 °C

**Figure 1.**
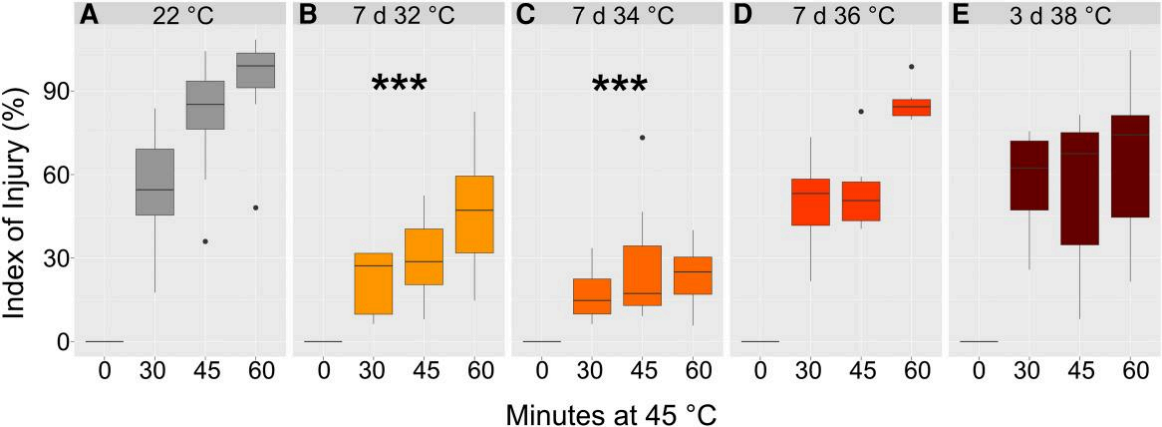
Heat tolerance of leaf tissue before and after acclimation. The index of injury (%) was based on the quantified electrolyte leakage of leaf tissue of plants grown at 22 °C **(A)** and heat acclimated **(B)** 7 d at 32 °C, **(C)** 7 d at 34 °C, **(D)** 7 d at 36 °C, and **(E)** 3 d at 38 °C. Asterisks indicate significant differences from the control, i.e. values at 22 °C (ANOVA and Tukey HSD post-hoc test, *** *P* < 0.001), *n* = 6. Center line, median; box limits, upper and lower quartiles; whiskers, 1.5 × interquartile range; points, outliers.

Susceptibility to heat stress was determined by quantifying the electrolyte leakage of leaf tissue to estimate membrane damage, described by the index of injury *I_d_*. Leaf tissue acclimated to 22 °C was damaged to ∼80% after 45 to 60 min of incubation in 45 °C water (Fig. 1A). Heat acclimation of leaf tissue after 7 d was most efficient for acclimation temperatures 32 and 34 °C which resulted in a significant reduction of *I_d_* values (ANOVA, Fig. 1, B and C). Acclimation at 36 (7 d) and 38 °C (3 d) resulted in a slight decrease of *I_d_* when compared to 22 °C plants, but the condition effect was not significant (Fig. 1, D and E). This was also in accordance with the growth phenotype of the plants after the heat treatment which indicated severe tissue damage at 36 and 38 °C, whereas for 32 and 34 °C, only an early induction of the inflorescence and slight yellowing of the leaves was observed (Supplementary Fig. S1).

**Figure 2.**
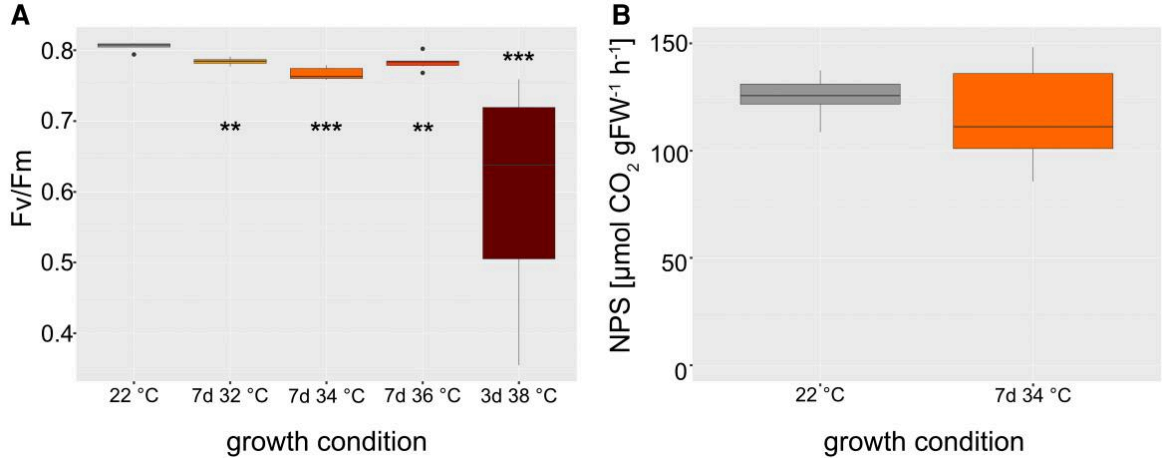
Photosynthesis under elevated temperature. **A)** Maximum quantum yield of photosystem II (*F_v_*/*F_m_*) as a function of acclimation duration and temperature. **B)** Rates of NPS. Asterisks indicate significant differences from the control (ANOVA and Tukey HSD post-hoc test, ** *P* < 0.01, *** *P* < 0.001), *n* = 6. Center line, median; box limits, upper and lower quartiles; whiskers, 1.5 × interquartile range; points, outliers. FW: fresh weight.

To reveal how elevated temperature affected photosynthetic efficiency and CO_2_ assimilation, the maximum quantum yield of photosystem II (*F_v_*/*F_m_*) was quantified together with rates of net photosynthesis (NPS) (Fig. 2). Under control conditions (22 °C), *F_v_*/*F_m_* values were >0.8 while they dropped significantly after 7 d at 32, 34, and 36 °C to values between 0.76 and 0.78. After 3 d at 38 °C, *F_v_*/*F_m_* showed the strongest decrease to a median of ∼0.64, and the data variance distinctly increased (Fig. 2A, dark red box). Due to the observation that leaf tissue acclimated most efficiently during 7 d at 34 °C, we also quantified CO_2_ assimilation under these conditions (Fig. 2B). Similar to *F_v_*/*F_m_*, NPS rates were slightly affected, but the decrease of the median under 34 °C was not significant.

### Heat response of the central carbohydrate metabolism

**Figure 3.**
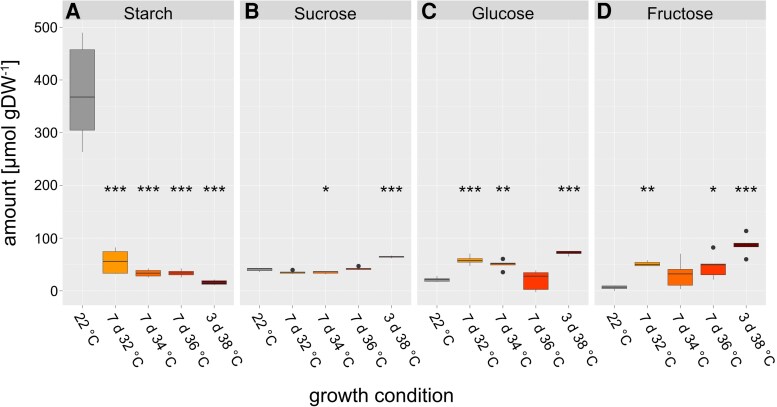
Metabolite amount per gram dry weight in control and acclimated plants. **A)** Starch amount in C6 equivalents. **B)** Sucrose amount. **C)** Glucose amount. **D)** Fructose amount. Asterisks indicate significant differences from the control (ANOVA and Tukey HSD post-hoc test, * *P* < 0.05, ** *P* < 0.01, *** *P* < 0.001), *n* ≥ 5. Center line, median; box limits, upper and lower quartiles; whiskers, 1.5 × interquartile range; points, outliers. DW: dry weight.

Starch amounts decreased significantly under elevated temperatures (Fig. 3A). Plants acclimated for 7 d at 32 °C had approximately 20% of starch found in the tissue of nonacclimated plants while amounts dropped even more for higher temperature regimes.

While, compared to nonacclimated plants, sucrose amounts also dropped in plants acclimated at 34 °C, they significantly increased approximately 1.5-fold after 3 d at 38 °C (Fig. 3B). Both glucose and fructose amounts were found to significantly increase during heat exposure with an exception at 36 °C for glucose and 34 °C for fructose (Fig. 3, C and D).

### Dynamics of subcellular sugar compartmentation under heat

Based on the finding that heat acclimation resulted in the highest tolerance after 7 d at 34 °C (Fig. 1, A and C), this condition was chosen for further detailed analysis of subcellular compartmentation of carbohydrates. The compartment-specific relative distribution of soluble sugars sucrose, glucose, and fructose was quantified before (22 °C) and after heat acclimation (7 d at 34 °C).

**Figure 4.**
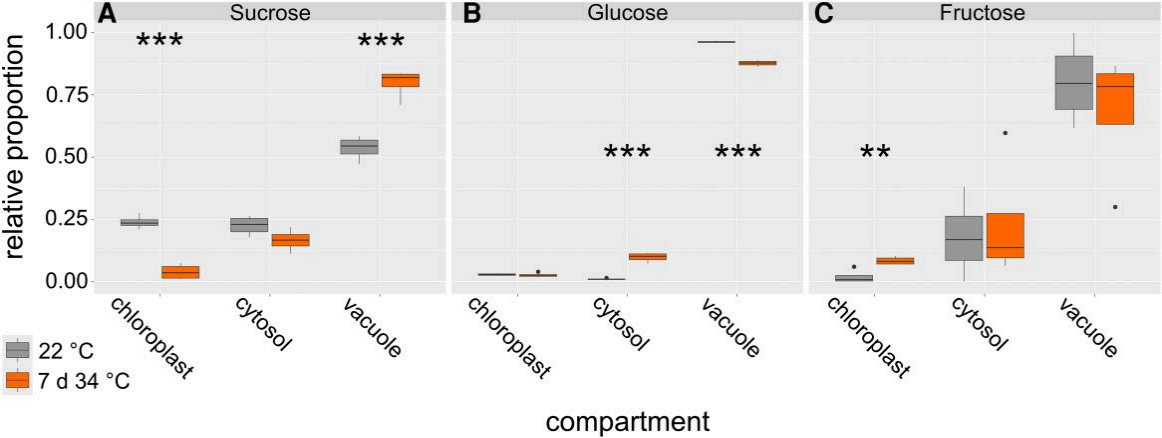
Effects of heat acclimation on subcellular distribution of soluble carbohydrates. Relative proportions for **(A)** sucrose, **(B)** glucose, and **(C)** fructose were resolved for chloroplasts, cytosol, and vacuole before (22 °C; gray boxes) and after heat acclimation at 7 d 34 °C (orange boxes). Asterisks indicate significance between both conditions (ANOVA and Tukey HSD post-hoc test; ** *P* < 0.01, *** *P* < 0.001), *n* = 4. Center line, median; box limits, upper and lower quartiles; whiskers, 1.5 × interquartile range; points, outliers.

For subcellular sucrose distribution, it was observed that heat induced a significant shift from chloroplasts to the vacuole (Fig. 4A). Due to heat exposure, the plastidial sucrose proportion decreased to less than 10%, while it increased to ∼80% in the vacuole. This shift was neither observed for glucose nor for fructose (Fig. 4, B and C). Under heat, glucose was slightly shifted from the vacuole to the cytosol, and fructose proportions remained relatively constant.

**Figure 5.**
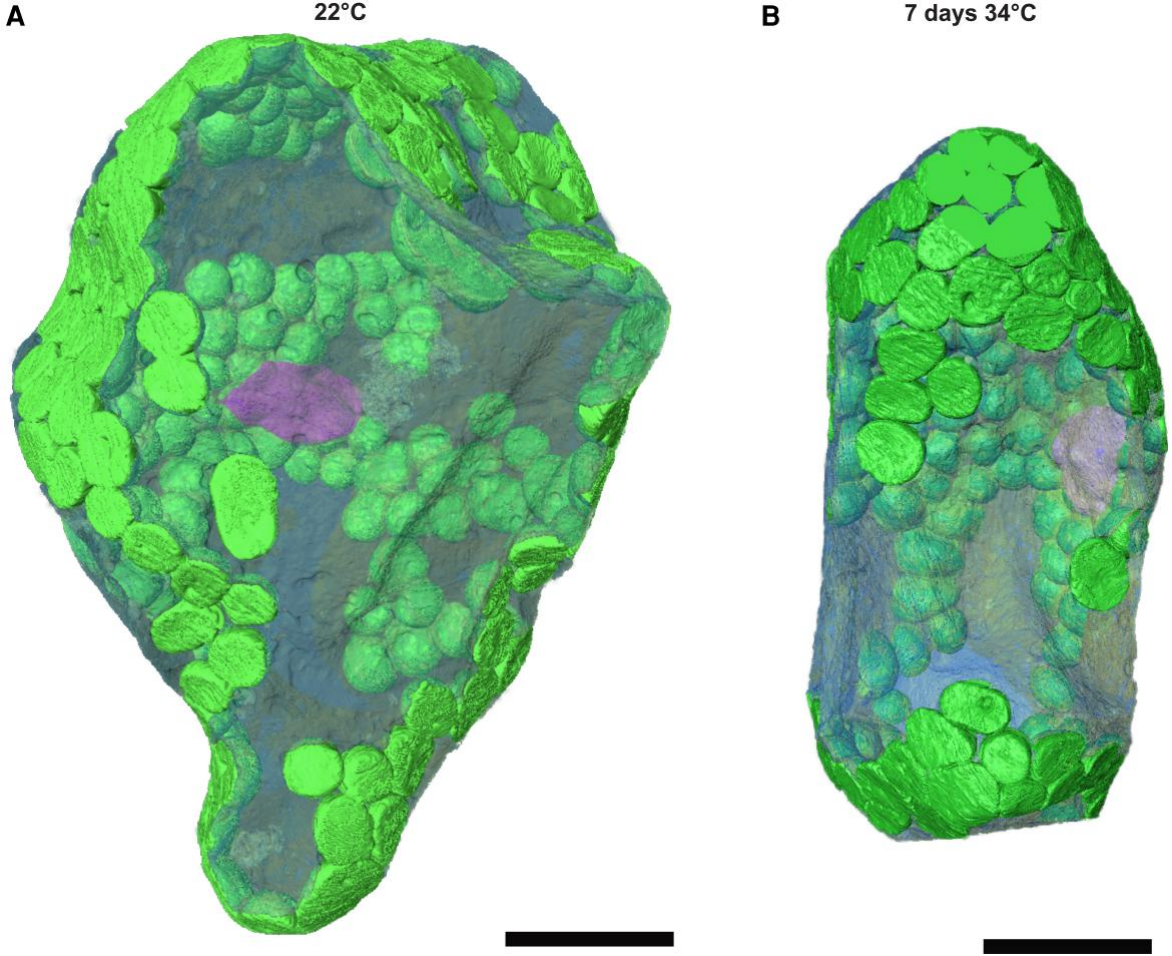
3D models of mesophyll cells. **A)** Control (22 °C) mesophyll cell. **B)** Heat-treated (7 d at 34 °C) mesophyll cell. Chloroplasts are shown in green, the nucleus in pink, and the vacuole in translucent blue. The cytosol encompassing other organelles such as mitochondria, Golgi apparatus, or endoplasmic reticulum, surrounding the vacuole and the chloroplasts is omitted in this view. Scale bars: 20 *µ*m.

The observed shifts in the relative proportion of sugars indicated a heat-induced regulation of subcellular compartmentation of carbohydrate metabolism. To reveal the effect of these relative shifts on compartment-specific metabolite concentrations, volumes of chloroplasts, cytosol, and vacuole were experimentally resolved by 3D SBF-SEM for mesophyll tissue at 22 and 34 °C (Fig. 5; Supplementary Fig. S2).

The evaluation of compartmental proportions revealed ∼14% chloroplasts, 3.5% cytosol, and ∼83% vacuole at 22 and 34 °C (Table 1). Together with the measured dry weight-to-fresh weight ratio and the average thickness of a leaf, fractions of compartment volumes in fresh leaves were determined. At 22 °C, the tissue volume per gram dry weight was about 1.5-fold higher than in heat-treated plants. Considering the estimated porosity from SBF-SEM analysis, this finally allowed for estimating the volume of cell material which was 10,932 mm³ gDW^−1^ at 22 °C and 8,267 mm³ gDW^−1^ at 34 °C. This information was combined with the compartmental proportions to estimate volumes of chloroplasts, cytosol, and vacuole with the dimension of mL gDW^−1^ (Table 1).

**Table 1.** Compartment volumes and leaf measures

Measurement	22 °C	34 °C
Proportion chloroplasts	13.89%	13.65%
Proportion cytosol	3.51%	3.63%
Proportion vacuole	82.6%	82.72%
Leaf height (mm) (*)	0.187 ± 0.005	0.138 ± 0.003
Leaf disc volume (mm^3^) (*)	9.415 ± 0.240	6.916 ± 0.130
Dry weight per leaf disc (mg) (NS)	0.622 ± 0.017	0.645 ± 0.074
Volume per gDW (mm^3^ g^−1^) (*)	15,131 ± 813	10,732 ± 1,616
Porosity (SBF-SEM)	27.8%	22.9%
Volume of gas space (mm^3^ gDW^−1^)	4,199.5 ± 225.6	2,465.5 ± 371.3
Volume cell material (mm^3^ gDW^−1^)	10,931.8 ± 587	8,266.5 ± 1,245
Estimated volume chloroplasts (mL gDW^−1^)	1.52 ± 0.08	1.13 ± 0.17
Estimated volume cytosol (mL gDW^−1^)	0.38 ± 0.02	0.30 ± 0.05
Estimated volume vacuole (mL gDW^−1^)	9.03 ± 0.49	6.84 ± 1.03

Proportions in percent were determined from SBF-SEM data. For leaf material at 22 °C, 13 cells were evaluated in the resin block. For leaf material at 7 d 34 °C, 28 cells were evaluated in the resin block. Leaf height was derived from light micrographs. Leaf disc volume was calculated from leaf height and punchout size (4 mm radius, i.e. 50.26 mm² area). Dry weight (DW) per leaf disc was determined by drying and weighing leaf discs. Volume per gram DW was calculated from leaf disc volume and dry weight. The porosity was acquired from SBF-SEM data and used to determine the volume of cell material per gram DW. This volume and the compartment proportions were used to estimate the volume of the different cell compartments per gram DW. Detailed calculations are collected in the supplements (Supplementary Table S2). NS: nonsignificant difference between both conditions. (*) significant difference between both conditions (ANOVA, *P* < 0.05). Means ± Se.

This revealed that, although relative proportions of analyzed compartments remained stable under heat, absolute compartment volumes decreased by about 25% after 7 d at 34 °C due to a reduced volume of leaf tissue per gDW.

The percentage of different cell types in a sampled leaf was then determined by light microscopy (Table 2). This analysis revealed approximately 57% mesophyll cells, 15% epidermal cells, 2% vascular bundle cells, and 26% porosity at 22 °C. During heat, mesophyll and epidermal cell proportions slightly increased to 58% and 16%, respectively. Vascular bundles did not change, whereas the porosity decreased to 24%.

**Table 2.** Percentage of cell types in a leaf section

Cell type	22 °C	34 °C
Palisade mesophyll (NS)	22.81 ± 2.68%	24.23 ± 1.24%
Spongy mesophyll (NS)	33.83 ± 2.68%	33.47 ± 1.11%
Epidermis (NS)	15.11 ± 1.18%	16.08 ± 1.89%
Vascular bundle (NS)	2.23 ± 0.95%	2.38 ± 1.44%
Porosity (NS)	26.01 ± 1.26%	23.84 ± 3.22%

The area of cell types was measured in light micrographs of semi-thin sections of embedded leaf material. Means ± Se, *n* = 4. NS: nonsignificant difference between both conditions (ANOVA, *P* > 0.05).

**Figure 6.**
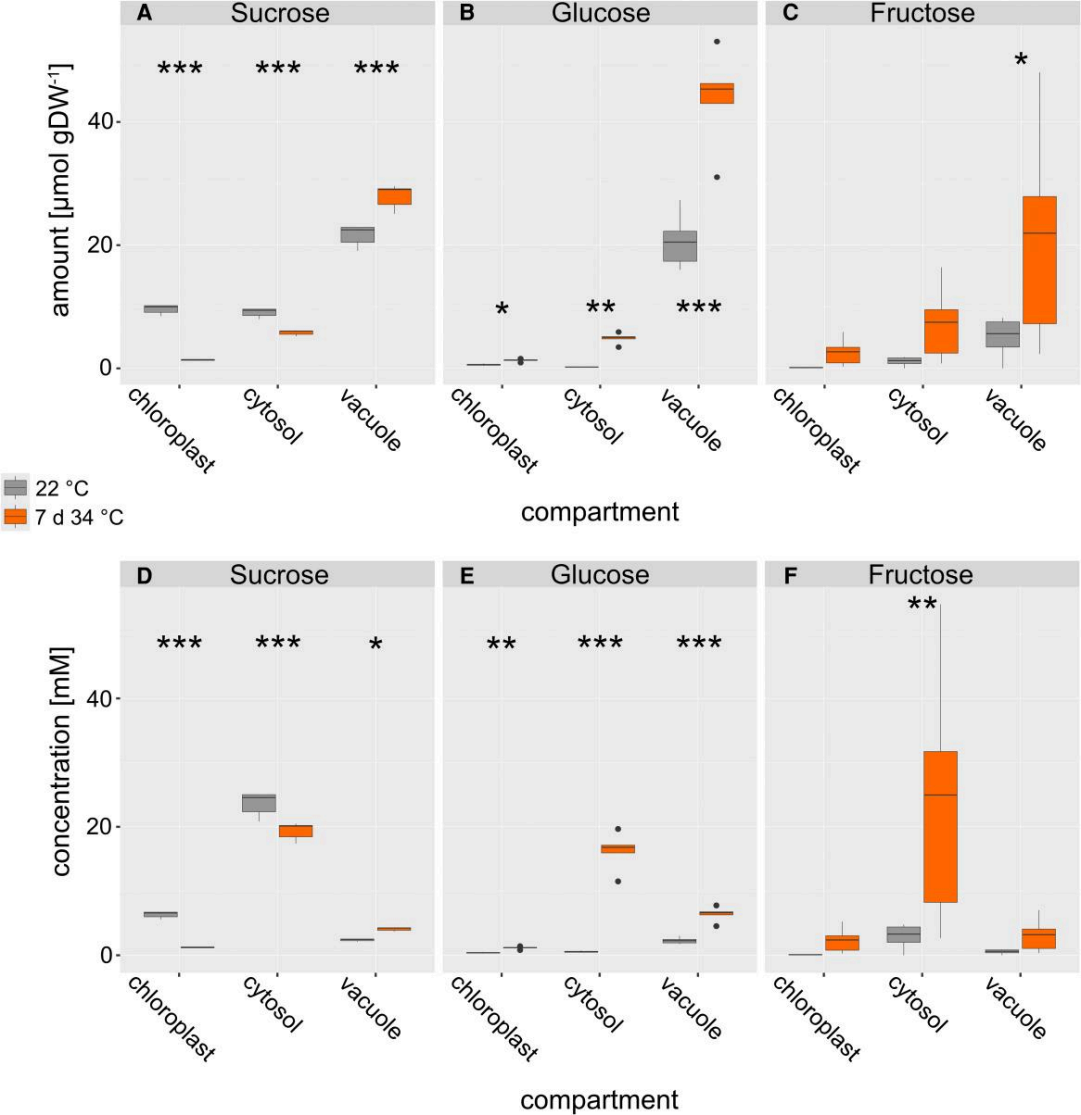
Compartment-specific sugar amounts and concentrations. Absolute amounts of sugars (*µ*mol gDW^−1^) in chloroplasts, cytosol, and vacuole are shown in the upper panels **(A** to **C)**, and concentrations (mm) are shown in the lower panels **(D** to **F)**. Gray boxes: 22 °C, orange boxes: 7 d at 34 °C. Asterisks indicate significance (ANOVA and Tukey HSD post-hoc test, * *P* < 0.05, ** *P* < 0.01, *** *P* < 0.001), *n* = 5. Center line, median; box limits, upper and lower quartiles; whiskers, 1.5 × interquartile range; points, outliers. DW: dry weight.

Combining the absolute sugar amounts with NAF-derived subcellular proportions revealed absolute sugar amounts of chloroplasts, cytosol, and vacuole at 22 °C and after 7 d at 34 °C (Fig. 6, A to C). Absolute sucrose amount differed significantly between both conditions across all compartments (Fig. 6A). The trend observed for relative proportions (see Fig. 4) was augmented, and cytosolic amounts now also differed significantly. Absolute amounts of glucose were significantly elevated due to heat acclimation across all compartments (Fig. 6B). In the vacuole, this contrasted the relative proportions which significantly decreased at 34 °C (compare Fig. 4B). However, due to a higher total amount of glucose under heat, this relative decrease still resulted in an absolute increase in the vacuolar compartment. Fructose was found to significantly accumulate in the vacuole which was not observed for relative proportions (compare Figs. 4C and 6C). As described for glucose, this was due to an increase in total fructose amounts. Yet, fructose dynamics were less significant than for glucose and sucrose.

Next, absolute compartment-specific sugar concentrations were derived from amounts normalized to estimated volumes of a mesophyll cell (Fig. 6, D to F). Interestingly, this emphasized accumulation effects in the cytosol which now also became significant for fructose (Fig. 6F). In general, while subcellular sugar amounts (in *μ*mol gDW^−1^) were highest in the vacuole, subcellular sugar concentrations (in mm) peaked in the cytosol which was due to the strong discrepancy of vacuolar and cytosolic volumes (see Table 1).

### Heat-induced dynamics of enzyme activities in sucrose metabolism

**Figure 7.**
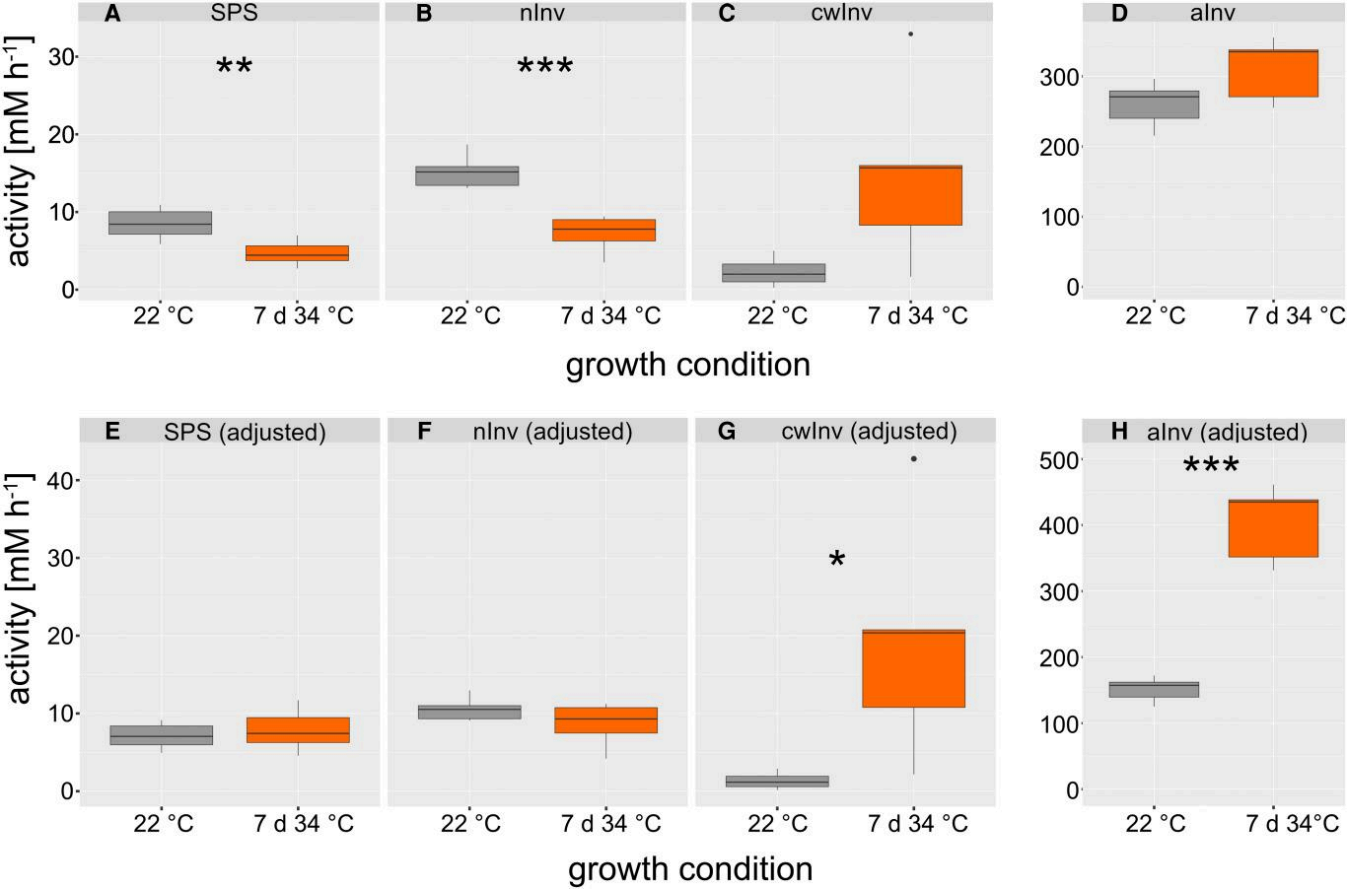
Activities of enzymes in sucrose metabolism. Invertase and SPS activity of plants grown at 22 °C (gray) and 34 °C acclimated plants (orange) were determined under substrate saturation (i.e. *V*_max_). The upper panel **(A** to **D)** represents experimentally quantified activities at temperature optimum (SPS: 25 °C; invertases: 30 °C). The lower panel **(E** to **H)** shows activities which were adjusted to the growth temperatures using the Arrhenius equation (see main text). Asterisks indicate significance in Student's *t*-test (* *P* < 0.05, ** *P* < 0.01, *** *P* < 0.001), *n* = 5. Center line, median; box limits, upper and lower quartiles; whiskers, 1.5 × interquartile range; points, outliers. nInv: neutral invertase; cwInv: cell wall-associated invertase; aInv: acidic invertase.

Based on the observation that heat significantly affected the compartmentation of sucrose and its hydrolytic cleavage products glucose and fructose, activities of central enzymes of sucrose biosynthesis (SPS) and cleavage (neutral, acidic, and cell wall-associated invertases; nInv, aInv, cwInv) were quantified before and after 7 d at 34 °C (Fig. 7). Activities were normalized to the volume of leaf cells, i.e. 10.93 mL gDW^−1^ at 22 °C and 8.26 mL gDW^−1^ at 34 °C. The activities of SPS and nInv decreased significantly under heat (Fig. 7, A and B), while cwInv and aInv activities increased nonsignificantly (Fig. 7, C and D).

Enzyme activities were quantified under substrate saturation at temperature optimum, which was 25 °C for SPS and 30 °C for invertases, respectively. To adjust those activities to the growth temperature of the plants, activation enthalpies were applied and used to solve the Arrhenius equation (Arrhenius 1889; Equation (1)).


(1) $$V_{\max,\mathrm{adj}}=C\cdot e^{-E_{a}/RT}$$

Here, *C* represents the Arrhenius factor, *E*_a_ is the activation energy, *R* is the gas constant, and *T* the temperature. The Arrhenius factor was estimated from experimentally determined enzyme activities at their temperature optimum as described earlier (Weiszmann et al. 2018; Supplementary Table S1). The adjustment to physiologically more relevant temperature regimes resulted in similar activities of SPS and nInv at 22 and 34 °C, respectively (Fig. 7, E and F). In contrast, adjustment resulted in significantly different activities of cwInv and aInv at 22 and 34 °C (Fig. 7, G and H). In summary, thermodynamic adjustment of enzyme activities revealed that both reactions located in the cytosol, SPS, and nInv, were efficiently stabilized under heat to maintain similar maximum enzyme activities as under 22 °C. In contrast, both enzymes located in acidic environments, i.e. apoplast (cwInv) and vacuole (aInv), significantly increased their maximum activities.

**Figure 8.**
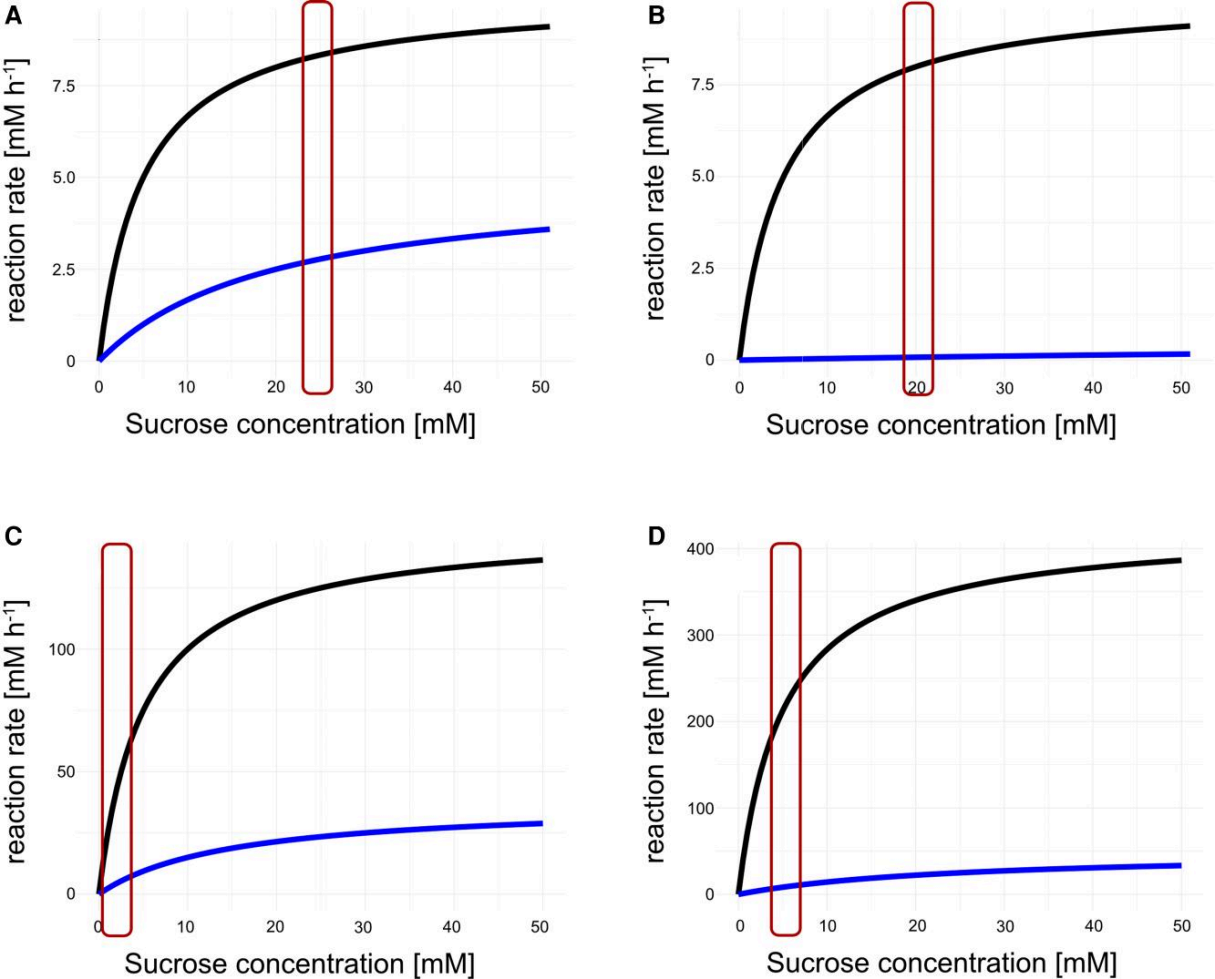
Simulation of Michaelis–Menten kinetics using subcellular metabolite concentrations and temperature-adjusted enzyme activities. **A)** Simulation of nInv catalyzed reaction at 22 °C, **B)** Simulation of nInv catalyzed reaction at 34 °C, **C)** Simulation of aInv catalyzed reaction at 22 °C, and **D)** Simulation of aInv catalyzed reaction at 34 °C. Black lines: no inhibition; Blue lines: combined competitive and noncompetitive inhibition by Frc and Glc, respectively. Red boxes indicate the relevant ranges of sucrose concentrations in vivo. Parameter settings are provided in the supplement (Supplementary Table S2). For simulations, the code for an R Shiny app is provided on GitHub: https://github.com/cellbiomaths/Shiny_MM_simulation).

Using the subcellular concentrations of sucrose, glucose, and fructose together with adjusted activities of enzymes, in vivo rates of cytosolic (nInv) and vacuolar (aInv) sucrose cleavage were estimated assuming Michaelis–Menten kinetics with competitive (Frc) and noncompetitive (Glc) inhibition (Sturm 1999, Fig. 8). Simulations showed that, under heat, cytosolic rates of sucrose cleavage were dramatically reduced due to the increased cytosolic hexose feedback inhibition of nInv (Fig. 8B). For aInv, which represents soluble acidic invertases with vacuolar localization, *V*_max_ was significantly increased at 34 °C which resulted in a maintenance of sucrose cleavage rates (Fig. 8, C and D) although also vacuolar concentrations of Frc and Glc increased under these conditions (see Fig. 6).

## Discussion

A changing temperature regime needs to be efficiently perceived and sensed by plants in order to initiate stress and acclimation response. Particularly, if temperature decreases or rises below or above critical values, molecular and physiological adjustments become essential to prevent irreversible cell and tissue damage. Adjustment and stabilization of photosynthesis and carbohydrate metabolism are central to temperature acclimation because a deflection of involved processes and pathways directly affects plant performance (Anderson et al. 1995; Qu et al. 2023). To quantify the heat tolerance of *A. thaliana* on levels of tissue structure and photosynthetic efficiency, electrolyte leakage assays were combined with measurements of *F_v_*/*F_m_* in the present study. While a 7-d acclimation period at 32 and 34 °C resulted in a significant reduction of leakage of leaf tissue when compared to nonacclimated plants, exposure to higher temperatures did not significantly improve tissue heat tolerance. Also, although *F_v_*/*F_m_* significantly dropped under all tested regimes of elevated temperature, it stabilized at ∼0.75 (except for the 38 °C treatment) which still indicated a relatively high efficiency of photosystem II. The functionality of photosynthesis was further proved by NPS rates which were stabilized after acclimation at 34 °C. However, pulse amplitude modulation (PAM) measurements were conducted on green chlorophyll-containing tissue while growth phenotypes at 36 °C already showed pale areas (see Supplementary Fig. S1), which are not reflected by these *F_v_*/*F_m_* values, and which were most probably a consequence of heat-induced senescence (Li et al. 2021). In summary, these findings suggest that heat exposure to temperatures between 32 and 34 °C significantly increases the heat tolerance of the plasma membrane of *A. thaliana*, which was not observed for higher temperatures. Heat-induced effects on *F_v_*/*F_m_* are robust across a wide range of acclimation temperatures which limits its applicability as a heat stress indicator. A limitation, which might affect the interpretation of heat-induced effects on photosynthesis and metabolism, was, however, the different developmental stages of 22 °C and 7 d heat-exposed plants. While plants at 22 °C were sampled and analyzed before bolting, heat-treated plants showed an inflorescence (see Supplementary Fig. S1). Such developmental differences might affect photosynthesis and metabolism (Rolland et al. 2006). However, as plant development directly depends on growth temperature (Ibañez et al. 2017), this could not be resolved in the chosen experimental setup, as a control experiment at 22 °C cannot be expected to result in the same developmental trajectory as under 32, 34, or 36 °C.

### Relative proportions of subcellular compartments and leaf tissues remain constant during heat acclimation

Heat can influence the architecture of plant cells and the different organelles within, varying depending on the duration and severity of the heat exposure. In chloroplasts, grana thylakoids were found to disorganize and unstack during heat exposure, especially at temperatures higher than 34 °C, and plastoglobuli numbers and sizes were found to increase with acclimation temperature (Supplementary Fig. S3). This has also been reported before, together with swelling of chloroplasts and mitochondria due to heat exposure (Gounaris et al. 1984; Vani et al. 2001; Zhang et al. 2010; Grigorova et al. 2012; Zhang et al. 2014; Zou et al. 2017; Jampoh et al. 2023). The reconstruction of leaf cells based on SBF-SEM in the present study revealed constant relative proportions of chloroplasts (13% to 14%), cytosol (3% to 4%), and vacuole (82% to 83%) before and after heat acclimation at 34 °C. Previously, similar proportions were reported and summarized for *Arabidopsis* epidermal pavement cells and mesophyll cells under ambient temperature (Tolleter et al. 2024). In this study, the authors derived a relative chloroplast volume of 9.4%, a cytosol volume of 3.8%, and a vacuole volume of 84.3%. Thus, while cytosol and vacuole directly correspond to the percentage determined in the present study, estimations of chloroplast volumes differed by 4% to 5% between both studies. Main reasons for this discrepancy might be that Tolleter and colleagues assembled data from various studies. This might imply (slightly) different growth conditions which might directly affect compartment-specific volumes in the observed range of discrepancy. Further, in the present study, leaf tissue was sampled at the end of the night which differs from studies compiled in the cell atlas (Tolleter et al. 2024). Nevertheless, although this indicates that chloroplast volumes might be over- or underestimated by up to 5%, the consistency across cytosolic and vacuolar volume estimations provides evidence for the applicability and robustness of the presented cellular volume information. In contrast, leaf disc volumes decreased by ∼30% in heat-acclimated plants while dry weight of the discs was not affected. This indicates a reduced (leaf) water content of heat-acclimated plants which might be due to higher transpiration rates under such conditions (Romero-Montepaone et al. 2021). Thus, while relative proportions of compartments remained constant, their absolute volumes decreased because of reduced total leaf tissue volume which suggests control of rather relative subcellular proportions than absolute compartment sizes. Similarly, also proportions of tissue types remained constant during heat acclimation (see Table 2). The proportions of palisade mesophyll, spongy mesophyll, epidermal tissue, and vascular bundles were similar before and after heat acclimation. We estimated porosity by 2D and 3D analysis to show the robustness of our results depending on the method. Both methods consistently showed a (nonsignificant) decrease in porosity during heat acclimation, ranging between 26% and 28% for leaf material at 22 °C and 23% and 24% after 7 d at 34 °C (see Tables 1 and 2). These estimates for plants grown at ambient temperature are in a similar range to those previously reported using microCT measurements of *A. thaliana* leaf discs (Dorca-Fornell et al. 2013; Lehmeier et al. 2017; Mathers et al. 2018).

In the present study, leaf cell structure was not analyzed for plants which were less efficiently heat acclimated, e.g. after 7 d at 36 °C. This leaves room for speculation if the observed proportional reduction of (sub)cellular structures and leaf tissue composition is a prerequisite for or a consequence of heat tolerance. Yet, as membrane remodeling was proven earlier to be essential for efficient heat acclimation (Kunst et al. 1989; Murakami et al. 2000; Shiva et al. 2020), the proportional reduction of compartment size might be accompanied by, or even facilitate, remodeling processes, e.g. changes in saturation degrees of membrane lipids and classes, due to reduced physical distances between membrane systems. Particularly, for vesicle trafficking and membrane contact-based lipid transfer (Shomo et al. 2024), such a reduction of physical distance might be beneficial. Finally, however, follow-up studies need to validate if the observed changes significantly affect remodeling and/or transport processes, or if the proportional reduction results in a maintenance of the homeostasis before heat exposure.

### Heat acclimation induces subcellular sugar allocation to stabilize sucrose metabolism during heat acclimation

A characteristic feature of eukaryotic cells is their compartmentation of metabolism which enables the separation and specification of pathways and their regulation (Hurry 2017). Carbohydrates are direct products of chloroplast-located photosynthetic CO_2_ assimilation which, following the fixation and reduction reactions of the CBBC, are allocated to different subcellular compartments. This results in metabolite dynamics which are difficult to trace because transport processes across membrane systems and enzymatic interconversions are versatile (Sweetlove et al. 2017; Pommerrenig et al. 2018). The method of NAF enables the immediate and persistent quenching of enzymatic reactions which conserves the metabolic status at the sampling timepoint (Gerhardt and Heldt 1984). In the present study, NAF revealed that, under heat, relative sucrose proportions are significantly decreased in the chloroplast and significantly increased in the vacuole. Together with the absolutely quantified metabolite amounts, this resulted in a significant increase of sucrose and glucose amounts in the vacuole. While such a vacuolar shift has also been reported before for cold acclimation (Knaupp et al. 2011; Fürtauer et al. 2016), the observed plastidial depletion of sucrose during heat acclimation contrasted findings made for low temperature (Nägele and Heyer 2013). Together with members of the raffinose family oligosaccharides (RFOs), sucrose was shown to protect liposomes in vitro against damage by fusion which suggested also a protective function in vivo, e.g. under drought or freezing temperatures (Hincha et al. 2003; Knaupp et al. 2011). In contrast to cold or freezing, membrane fluidity is increased under heat which may not need the accumulation of sucrose or raffinose in the chloroplast to prevent fusion of thylakoid membranes. Instead, when subcellular sugar concentrations were calculated from total sugar amounts and estimated compartment volumes, cytosolic sucrose concentrations became highest under both 22 and 34 °C due to the comparatively low cytosolic volume. For glucose and fructose, a significant heat-induced cytosolic increase was observed which immediately raised the hypothesis of hexose accumulation due to affected invertase activity. Quantifying cytosolic neutral invertase activity revealed a stabilized activity between 22 and 34 °C, which became evident after normalization to leaf tissue volumes and correction for thermodynamic effects by applying the Arrhenius equation. Hence, although previous studies have shown that several proteins involved in carbon assimilation and metabolism are downregulated under heat stress, this might not necessarily result in a downregulation of the in vivo reaction rates due to additional thermodynamic effects (Ahsan et al. 2010). Vacuolar invertase activity was significantly elevated by heat exposure. This led to the hypothesis that sucrose is transported along its concentration gradient from the cytosol to the vacuole where it is cleaved hydrolytically to release glucose and fructose. Such metabolite transport across the tonoplast might be facilitated by monosaccharide transporters as explained and outlined before (see e.g. Pommerrenig et al. 2018). Data from the present study suggests a concentration gradient of sucrose from the cytosol to the vacuole of mesophyll cells. While this gradient remained similar under 22 °C and after 7 d at 34 °C, cytosolic glucose and fructose concentrations rose significantly under heat. Further, sucrose concentrations in the chloroplast dropped significantly under heat (see Fig. 6, D to F). It remains speculation in the present study, but such dynamics of plastidial and cytosolic sugar concentrations might result in, or be a consequence of, dynamics of membrane transport. Previous studies have shown and summarized potential candidates of sugar transporters across intracellular membrane systems, which strongly affect plant stress response, acclimation, and development (Klemens et al. 2013; Guo et al. 2023; Valifard et al. 2023). A decreasing sucrose concentration in the chloroplast might suggest a changing expression or activity of transport proteins located in the chloroplast envelope. Interestingly, the chloroplast sucrose exporter pSUT has previously been found to critically affect flowering and cold response (Patzke et al. 2019). Further, flowering has earlier been shown to be potently induced by elevated growth temperature (Balasubramanian et al. 2006). While, in the present study, we did not statistically uncouple plant development from heat exposure, it became obvious from the phenotypes that heat exposure at least temporally fell together with flowering (see Supplementary Fig. S1). As it was shown that suppressed pSUT expression results in impaired inflorescence development (Patzke et al. 2019), this further supports the hypothesis that pSUT expression and activity might critically affect development and acclimation under heat by modifying the sucrose concentration gradient across the chloroplast envelope.

Dynamics of both subcellular metabolite concentrations and enzyme activities result in dynamics of enzymatic reaction rates if metabolites act as substrates, products, or regulatory effectors of enzymes. The simulation of reaction rates catalyzed by neutral and acidic invertases showed that, under heat, neutral invertase flux became very low which was caused by the accumulation of hexoses in the cytosol which act as inhibitors (Sturm 1999). While also acidic invertase flux was affected by the heat-induced accumulation of hexoses in the vacuole, a strong and significant increase of *V*_max_ counteracted this inhibition resulting in similar reaction rates at 22 and 34 °C (see Fig. 8). Similarly, for plant cold acclimation, it was discussed earlier that temperature-induced shift of sucrose cleavage into the vacuolar compartment might stabilize metabolism and photosynthesis (Weiszmann et al. 2018). Together with the observed heat-induced depletion of plastidial sucrose concentration, these findings suggest that sucrose transport across the chloroplast envelope and tonoplast is coordinated with subcellular invertase activities during heat exposure to stabilize cytosolic sucrose concentration which plays a central role in regulation of photosynthesis, energy metabolism, and sink–source interactions (Ruan 2014).

## Materials and methods

### Plant material

Plants of *A. thaliana*, Columbia-0 (Col-0), were grown at 22 °C in the greenhouse (∼100 to 125 *µ*mol m^−2^ s^−1^, ∼12 h/12 h light/dark, 60% to 70% rel. humidity). After 4 weeks of growth, at the initial bolting stage, the control plants were harvested. For heat acclimation, plants were transferred to the respective temperature at a light intensity of 100 *µ*mol m^−2^ s^−1^, 12 h/12 h light/dark, 60% to 70% rel. humidity, and watered regularly to prevent drought stress. Temperature treatment included 32, 34, 36, and 38 °C. After 7 d of heat treatment, the plants were harvested. For 38 °C, harvesting took place after 3 d of heat treatment due to the high mortality of the plants beyond that timepoint. Harvesting for analysis of metabolism took place at mid-day, i.e. after 6 h in light, by cutting the plants at the hypocotyl and plunge-freezing them in liquid nitrogen. The inflorescences, if established, were excluded from analysis. The frozen plant material was stored at −80 °C until it was ground to a fine powder and freeze-dried. Samples for electron microscopy were collected at the end of the night to prevent starch accumulation in the chloroplasts.

### Electrolyte leakage

An electrolyte leakage assay was performed on intact leaves from control plants and acclimated plants. Two leaves per sample and 6 samples per temperature step (*n* = 6) were cut from 1 plant and fully submerged in 7 mL of distilled H_2_O. The samples were heated to 46 °C for 0, 30, 45, and 60 min. They were cooled down and shaken overnight at room temperature. Then, 1 mL of water was taken from the samples, diluted 1:4 in H_2_O and initial electrical conductivity EC_initial_ (*µ*S cm^−1^) was determined. Then, the samples were heated to 95 °C for 1 h, and total electrical conductivity EC_total_ (*µ*S cm^−1^) was determined again. The index of injury *I_d_* was calculated individually for the different timepoints with the fractional release of electrolytes from nonheated and heated samples, *R*_0_ and *R_t_* (Equations (2) and (3); Flint et al. 1967).

The index of injury *I_d_* from exposure to temperature


(2) $$I_{d}\;=\;100\frac{R_{t}\;-\;R_{0}}{1-R_{0}}$$

Fractional release of electrolytes from nonheated *R*_0_ and heated sample *R_t_*


(3) $$R_{0},\;R_{t}\;=\;\frac{EC_{\mathrm{initial}}}{EC_{\mathrm{total}}\;}$$

### Chlorophyll fluorescence and gas exchange measurements

To evaluate the effect of prolonged heat exposure on photosystem II, maximum quantum yield (*F_v_*/*F_m_*) was recorded by supplying a saturating light pulse after 15 min of dark adaptation at 22 °C (WALZ JUNIOR-PAM; Heinz Walz GmbH, Germany). Gas exchange was quantified at 22 and 34 °C using the GFS-3000 with measuring head 3010-S (Heinz Walz GmbH, Germany).

### Metabolite quantification

The amounts of carbohydrates were determined as described before (Kitashova et al. 2023). Soluble carbohydrates were extracted twice with 400 *µ*L 80% ethanol at 80 °C for 30 min. The supernatants were combined and dried for sugar analysis. The pellet was used for starch quantification by amyloglucosidase digestion and photometric detection of glucose equivalents by a coupled glucose oxidase/peroxidase/*o*-dianisidine reaction. Sucrose content was determined by an anthrone assay, whereas glucose concentration was determined by a coupled hexokinase/glucose-6-phosphate dehydrogenase assay, utilizing absorption measurement of produced NADPH + H ^+^ . Fructose quantification followed glucose quantification by the addition of phosphoglucose isomerase (PGI) to the reaction buffer.

### Invertase and SPS activity measurements

The activities of vacuolar (acidic), cytosolic (neutral), and cell wall-bound invertases, as well as of SPS, were quantified as described earlier with slight modifications (Nägele et al. 2012; Kitashova et al. 2023). Invertases were extracted on ice in extraction buffer (50 mm HEPES-KOH, pH 7.5, 5 mm MgCl_2_, 2 mm ethylenediaminetetraacetic acid (EDTA), 1 mm phenylmethylsulfonylfluoride, 1 mm dithiothreitol (DTT), 0.1% (v/v) Triton-X-100, 10% (v/v) glycerol). After centrifugation, the supernatant was analyzed for vacuolar and cytosolic invertases, whereas the pellet was resuspended in an extraction buffer to analyze the cell wall-bound invertase activity. After the incubation of the supernatant at 30 °C in an acidic reaction buffer (20 mm sodium acetate, pH 4.7, 100 mm sucrose) for vacuolar and cell wall invertase measurements, or a neutral reaction buffer (20 mm HEPES-KOH, pH 7.5, 100 mm sucrose) for cytosolic invertase, the solution was neutralized with 1 m NaH_2_PO_4_, heated to 95 °C to stop the enzymatic reaction, and centrifuged. The glucose content in the supernatant was quantified photometrically by a coupled glucose oxidase/peroxidase/*o*-dianisidine reaction.

SPS was extracted in the extraction buffer (50 mm HEPES-KOH, pH 7.5, 20 mm MgCl_2_, 1 mm EDTA, 2.7 mm DTT, 10% (v/v) glycerol, and 0.1% (v/v) Triton-X-100) on ice and centrifuged. The supernatant was incubated with a reaction buffer (50 mm HEPES-KOH, pH 7.5, 15 mm MgCl_2_, 3 mm DTT, 35 mm uridine diphosphate [UDP]-glucose, 35 mm fructose-6-phosphate, and 140 mm glucose-6-phosphate) at 25 °C and the reaction was stopped by adding 30% KOH and heating the solution to 95 °C. Sucrose was quantified photometrically with an anthrone assay.

### Nonaqueous fractionation

NAF was performed as described previously (Fürtauer et al. 2016; Hernandez et al. 2023). Briefly, 10 to 15 mg of lyophilized plant material was suspended in 1 mL of heptane (C_7_H_16_; “7H”) -tetrachlorethylene (C_2_Cl_4_; “TCE”) mixture with a density of *ρ* = 1.35 g cm^−3^ and sonicated on ice in 30 s pulses with pauses of 1 min for a total of 20 min (Hielscher UP200St Ultrasonic Homogenizer, 170 W, 100% power setting; Hielscher Ultrasonics GmbH, Teltow, Germany). The sonicated material was centrifuged for 20 min at 4 °C and 20,000*×g*. The supernatant was stored on ice and the pellet was suspended in a 7H-TCE mixture of higher density and sonicated for 10 s to facilitate the dissolving of the pellet. Subsequently, the material was centrifuged, and the new pellet was suspended in a higher density 7H-TCE mixture. This process was repeated with mixtures of increasing density, ranging from *ρ* = 1.35 to 1.6 g cm^−3^. The fractions were split equally and dried in a vacuum desiccator. Pellets were stored at −20 °C until subsequent analysis. One aliquot per sample was used for photometric marker enzyme measurements for vacuole, cytosol, and chloroplast and the other for photometric evaluation of sugar content.

### Marker enzyme activities

For photometric measurement of marker enzyme activities, the dried pellets of fractions were suspended in 750 *µ*L extraction buffer (50 mm Tris-HCl, pH 7.3, 5 mm MgCl_2_, 1 mm DTT), incubated on ice for 10 min and centrifuged at 4 °C and 20,000*×g* for 10 min. The supernatant was used for enzyme activity quantification. Plastidial pyrophosphatase was used as a marker enzyme for the chloroplast, cytosolic UDP glucose pyrophosphorylase (UGPase) for the cytosol, and vacuolar acidic phosphatase for the vacuole (Fürtauer et al. 2019). Plastidial pyrophosphatase was assayed as described earlier (Jelitto et al. 1992) and inorganic phosphate was detected by molybdenum blue reaction (Murphy and Riley 1962). UGPase was quantified photometrically as described before (Zrenner et al. 1993). Acidic phosphatase from the vacuole was measured according to Boller and Kende (1979), with some modifications. The assay buffer consisted of 125 mm sodium acetate and 0.125% Triton-X 100 and was adjusted to pH 4.8 with acetic acid, whereas the substrate for the detection was composed of 1 mg mL^−1^ 4-nitrophenylphosphate in the assay buffer.

### Sample fixation for microscopy

Several leaves per plant were fixed for microscopy. The plants were harvested at the end of the dark period to minimize starch content in the plastids, which improves the visibility of thylakoid membranes. The leaves were cut into 1 mm^2^ pieces in fixation buffer (75 mm cacodylate, 2 mm MgCl_2_, pH 7.0) supplemented with 2.5% glutaraldehyde and stored at 4 °C for several days until further processing.

### Light and transmission electron microscopy

For light and transmission electron microscopy (TEM), fixation was carried out as described before (Garcia-Molina et al. 2021). After postfixation with 1% (w/v) OsO_4_, the samples were contrasted en bloc with 1% (w/v) uranyl acetate in 20% acetone, dehydrated with a graded acetone series and embedded in Spurr's resin of medium rigidity (Spurr 1969). For TEM, ultrathin sections of approximately 60 nm were contrasted with lead citrate (Reynolds 1963) and examined with a Zeiss EM 912 transmission electron microscope with an integrated OMEGA energy filter, operated at 80 kV in the zero-loss mode (Carl Zeiss AG, Oberkochen, Germany). Images were acquired with a 2k × 2k slow-scan CCD camera (TRS Tröndle Restlichtverstärkersysteme, Moorenweis, Germany). For light microscopy, semi-thin sections of 1 *µ*m were examined with a Zeiss Axiophot microscope and a SPOT Insight camera.

### Serial block-face scanning electron microscopy

For SBF-SEM, 1 mm^2^ pieces of *A. thaliana* leaves were fixed and stained following a protocol based on Hua et al. (2015). In brief, with intermittent washing steps, the samples were fixed as described above, treated with 2% OsO_4_ + 1.5% potassium ferrocyanide on ice, with thiocarbohydracid solution at RT, again 2% OsO_4_, 1% uranyl acetate, then lead aspartate at 60 °C. Following an ascending ethanol and acetone series, the samples were embedded in epon resin hard 812, mounted on aluminum stubs with conductive glue, trimmed to 500 *µ*m cubes, and sputtered with 20 nm gold.

Serial sectioning and imaging took place on a ThermoFisher Apreo VS block-face scanning electron microscope (Thermo Fisher Scientific Inc., Waltham, USA) in low vacuum at 2.1 kV, 100 to 200 pA and a pixel dwell time of 3 *µ*s. Digital image stacks (grayscale, 8 bit) of 8,192 × 8,192 pixels at 20 nm pixel size and 40 nm cutting thickness were generated and postaligned with Fiji, with stack 1 amounting to 1,872 planes and stack 2 amounting to 1,450 planes.

### Segmentation of cellular compartments

The SBF-SEM image stacks were processed with the Amira Pro software (Versions 2019-2024.1, Thermo Fisher Scientific). In a fraction of the 2 datasets, chloroplasts, vacuole, cytoplasm, and nucleus were segmented manually with a graphic tablet. Smaller organelles such as mitochondria, peroxisomes, Golgi apparatus, and endoplasmic reticulum were not segmented but were included in the cytoplasm material. The Python Deep Learning environment of Amira was utilized for automated segmentation of the datasets with the manually segmented data serving as ground truth. Learning settings were adjusted between training steps and are summarized in Supplementary Table S3. The resulting labels were checked for mislabeling and corrected manually.

### Data analysis and calculation of subcellular volumes

Subcellular sugar content was correlated to the marker enzyme measurements with the “NAFalyzer” app to estimate subcellular sugar concentrations relative to sample dry weight (Hernandez et al. 2023). The relative distribution of marker enzyme activities was correlated with metabolite abundances. For both conditions, i.e. 22 °C and 7 d 34 °C, plastidial marker enzymes showed a peak at the lowest density, *ρ* = 1.35 g cm^−3^. In contrast, vacuolar marker enzyme activities peaked at the highest density, i.e. *ρ* = 1.60 g cm^−3^. The distribution of the cytosolic marker was similar across all fractions but rather peaked at *ρ* = 1.40 to 1.45 g cm^−3^ (Supplementary Fig. S4). Hence, although each fraction contained a mixture of marker enzyme activities, these different distributions of marker enzymes across all fractions allowed for a reliable estimation of compartment-specific metabolite concentrations (Gerhardt and Heldt 1984).

Leaf discs of 4 mm radius were punched out and subsequently dried to quantify fresh and dry weight (*n* = 45). Average leaf height (*n* ≥ 22) from light micrographs was multiplied with disc size to determine the leaf disc volume. The volume-to-dry weight ratio was calculated and corrected for gas space, i.e. porosity and percentage of the segmented SBF-SEM dataset. The volume per dry weight ratio of plastids, cytosol, and vacuole was calculated from the percentages of the compartments from the SBF-SEM dataset and further combined with the subcellular sugar amount to calculate the absolute subcellular sugar concentrations in mm (Supplementary Tables S4 and S5). The percentage of cell types in the leaf was measured in light microscopic images of semi-thin sections of embedded leaf material (*n* = 4). The area of different cell types was measured in Fiji with the help of the standard measurement tool and a graphic tablet. Image analysis of electron and light micrographs was carried out with Fiji. Data analysis and statistics were carried out with R (The R Project for Statistical Computing; https://www.r-project.org) and Microsoft Excel (https://www.microsoft.com). The development of the R shiny app for the simulation of Michaelis–Menten kinetics was supported by ChatGPT (October 2024). The code is provided via GitHub: https://github.com/cellbiomaths/Shiny_MM_simulation.

## Supplementary Material

kiaf117_Supplementary_Data

## Data Availability

Data is provided in the supplements and, on request, by the corresponding authors. The code of the R Shiny app for kinetic simulations is provided on GitHub, https://github.com/cellbiomaths/Shiny_MM_simulation.

## Supplementary data

The following materials are available in the online version of this article.

**Supplementary Table S1.** Temperature correction of enzyme activities.

**Supplementary Table S2.** Enzyme parameters for invertase simulations.

**Supplementary Table S3.** Settings for the “DL Training Segmentation 2D” module in Amira Pro for AI-based segmentation.

**Supplementary Table S4.** Data and calculation of subcellular volumes.

**Supplementary Table S5.** Total and subcellular metabolite amounts, relative distributions, and concentrations.

**Supplementary Figure S1.** Plant phenotypes after heat treatment.

**Supplementary Figure S2.** SBF-SEM imaging of plant tissue.

**Supplementary Figure S3.** Light and electron micrographs of control and heat-treated leaves of *Arabidopsis thaliana*.

**Supplementary Figure S4.** Relative distribution of marker enzyme activities across nonaqueous density fractions.

## Dive Curated Terms

The following phenotypic, genotypic, and functional terms are of significance to the work described in this paper:

metabolite CHEBI: CHEBI:25212

UDP Gramene: Uridine diphosphate
UDP Araport: Uridine diphosphate
