# Supplementary material for: Subcellular plant carbohydrate metabolism under elevated temperature
Source: Plant Physiol. 2025 Apr 16;198(3):kiaf117. doi: 10.1093/plphys/kiaf117 (PMC12225672; doi:10.1093/plphys/kiaf117)
Supplement: kiaf117_Supplementary_Data [file kiaf117_supplementary_data.zip › Supplementary Data.pdf]

Supplementary Data

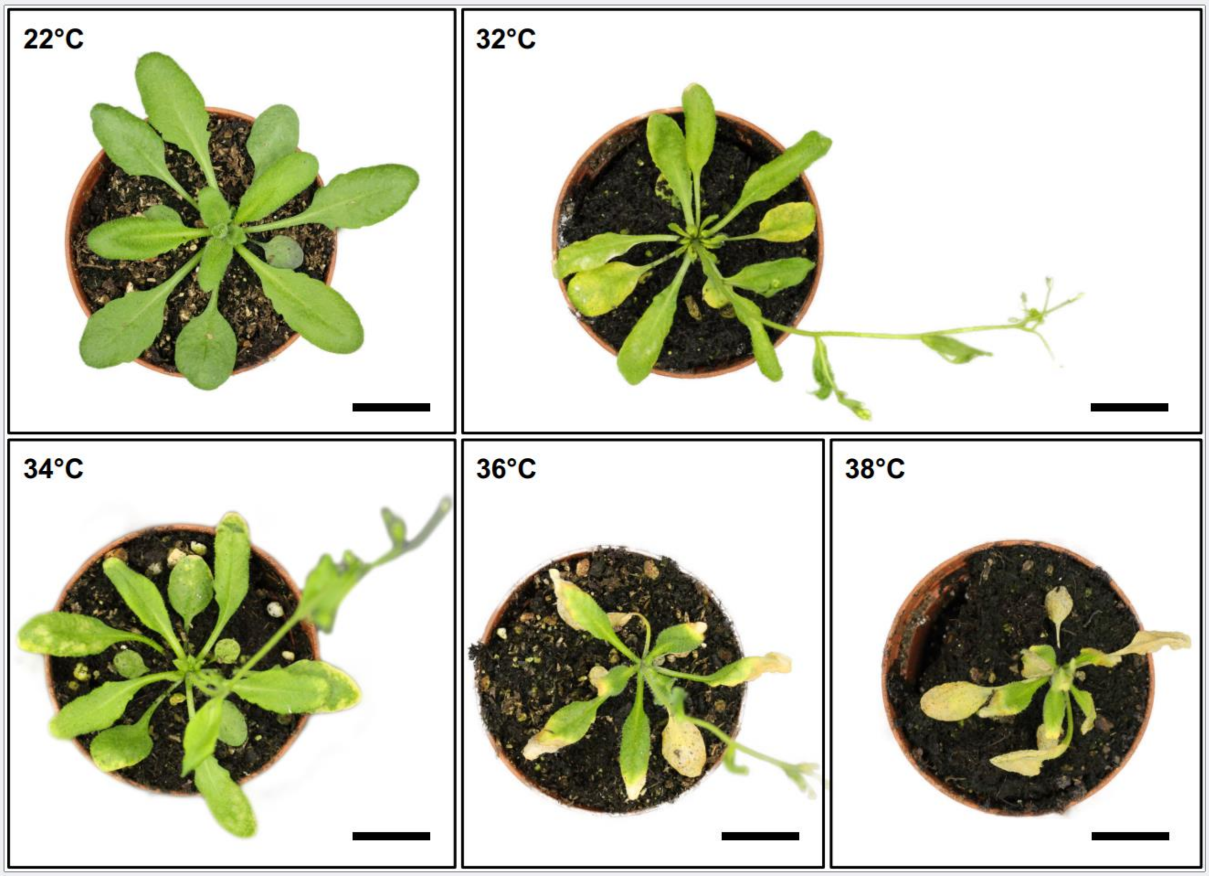

Supplementary Figure S1. Plant phenotypes after heat treatment. Control (22 °C), 7 days 32 °C, 7 days 34 °C, 7 days 36 °C and 3 days 38 °C. Treatment with 32 °C to 36 °C resulted in yellowing of leaves in increasing severity and early induction of inflorescence. Treatment with 38 °C resulted in a low survival rate beyond 3 days of heat exposure. Surviving plants showed heavy yellowing of leaves and no induction of inflorescence. Images were digitally extracted for comparison. Scalebars: 2 cm.

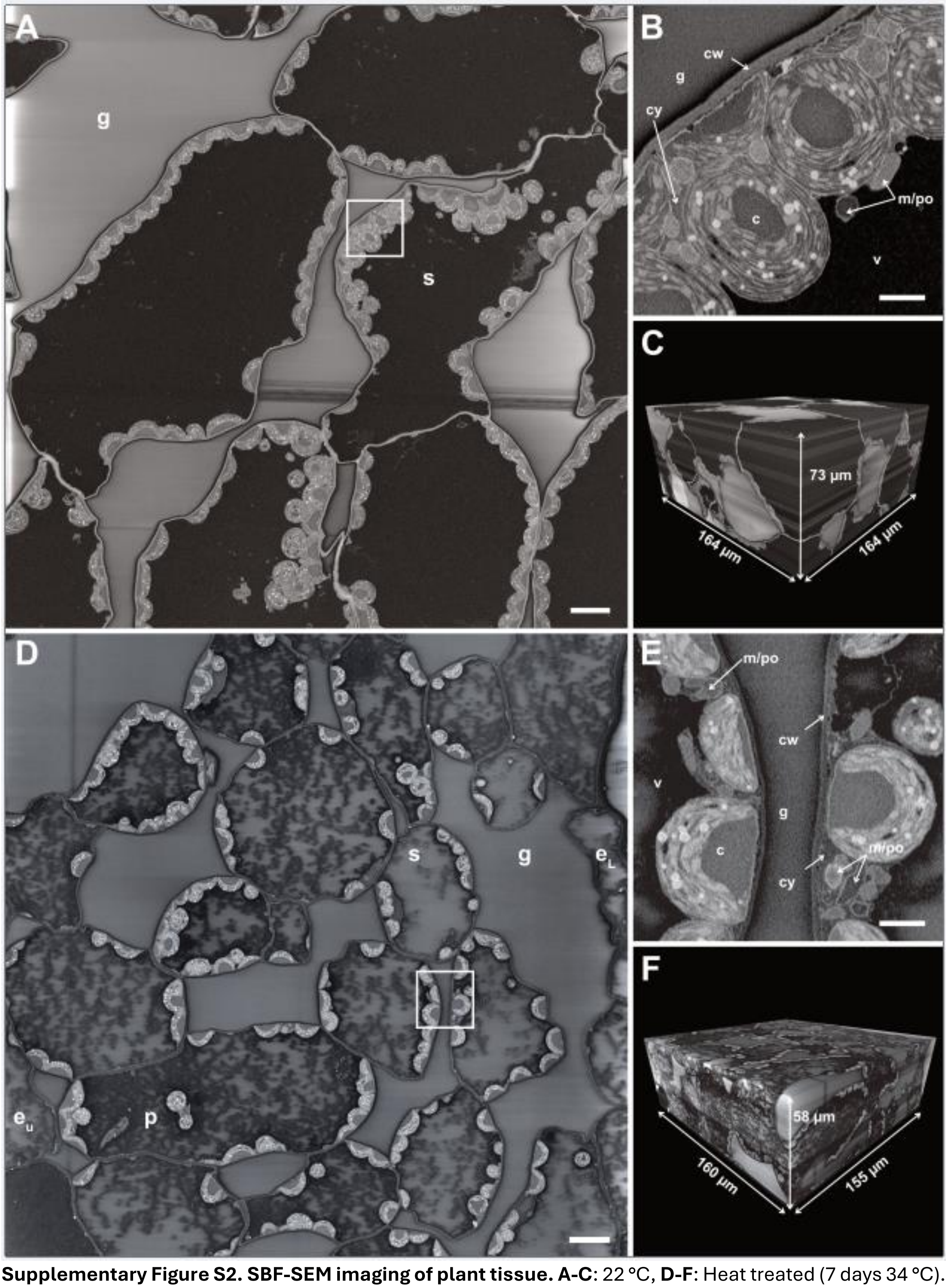

Supplementary Figure S2. SBF-SEM imaging of plant tissue. A-C: 22 °C, **D-F**: Heat treated (7 days 34 °C). **A+D**: Slice of sample block; e_U = upper epidermis, e_L = lower epidermis, g = gas space, p = palisade mesophyll cell, s = spongy mesophyll cell. Scalebar: 10µm. **B+E**: Magnification of the area marked with a square in panels **A** and **D**, c = chloroplast, cw = cell wall, cy = cytosol, g = gas space, m/po = mitochondria or peroxisome, v = vacuole. Scalebar: 2µm. **C+F**: Complete sample block with dimensions in µm.

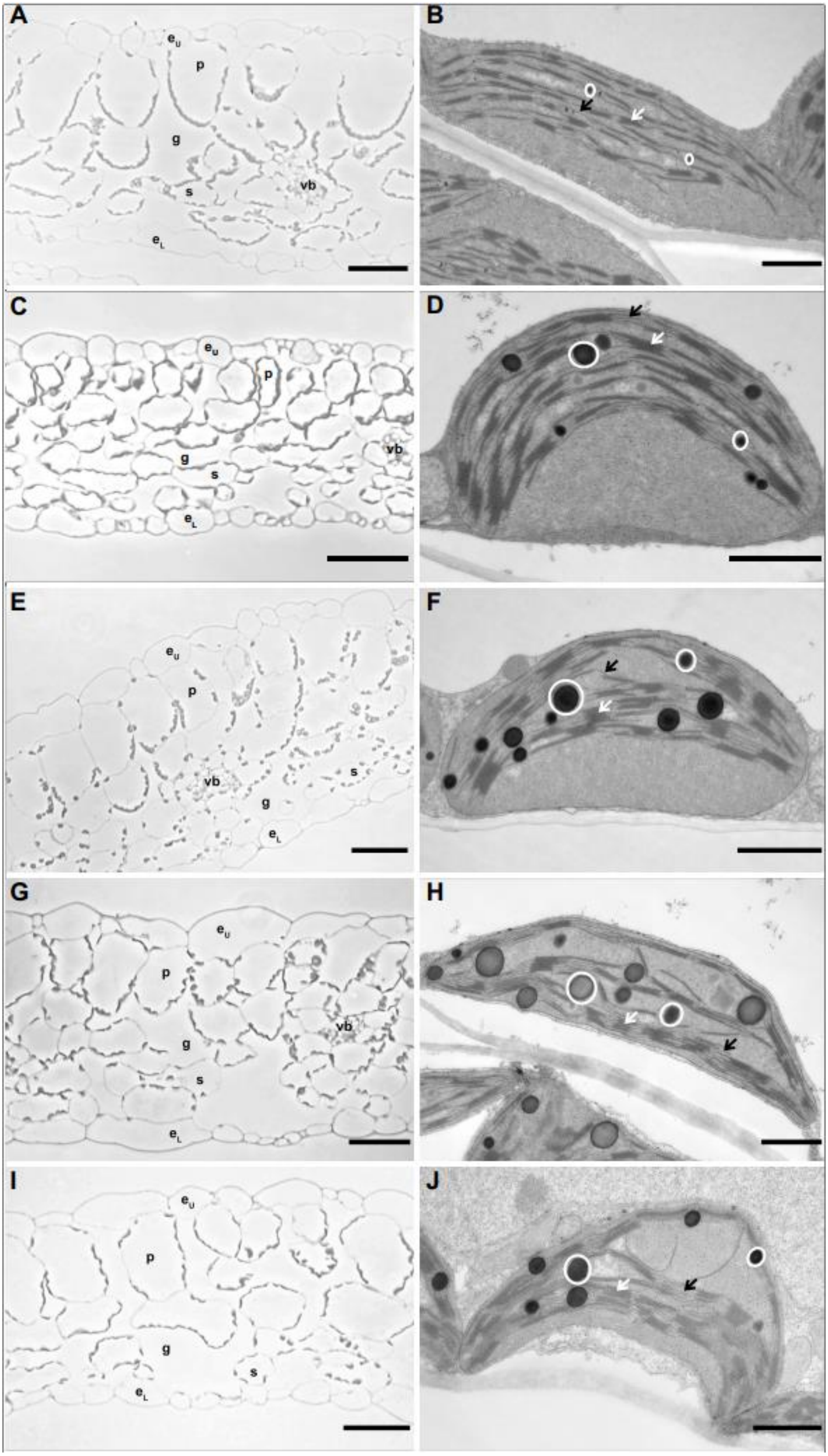

A
eU
p
g
s
vb
eL
B
C
eU
p
vb
g
s
eL
D
E
eU
p
vb
s
g
eL
F
G
eU
p
vb
g
s
eL
H
I
eU
p
g
s
eL
J

Supplementary Figure S3. Light and electron micrographs of control and heat-treated leaves of *Arabidopsis thaliana*. A, C, E, G, I: Light microscopic images of semi thin sections of embedded leaf material; e_U: upper epidermis, e_L: lower epidermis, g: gas space, p: palisade mesophyll, s: spongy mesophyll, vb: vascular bundle, scalebar = 50 µm. **B, D, F, H, J:** Transmission electron micrographs of chloroplasts in ultrathin sections of embedded leaf material; white circles: plastoglobules, white arrows: grana thylakoids, black arrows: stroma thylakoids, scalebar = 1 µm. **A**: leaf section of control plant (22 °C). The leaf section shows typical architecture for *A. thaliana* leaves, the palisade mesophyll constitutes the upper layer of the mesophyll tissue, the spongy mesophyll the lower layer, vascular bundles are located in the middle, mostly in the spongy mesophyll; upper and lower epidermis both contain stomata and enclose the mesophyll tissue. **B:** Chloroplast of control plant (22 °C). The thylakoid membranes are arranged regularly and parallel to the vacuole facing side of the chloroplast, the plastoglobules are small and evenly distributed between the thylakoid membranes. **C:** Leaf section after 7 days at 32 °C; the tissue is packed more densely, and the cells are in average smaller than in the control leaf. **D:** Chloroplast after 7 days of 32 °C; the thylakoid membranes are still arranged similarly to the control, but the grana stacks are higher; the plastoglobules are larger than in the 22 °C chloroplast. **E:** Leaf section after 7 days of 34 °C; the leaf architecture is comparable to 32 °C, the tissue is more densely packed than in the 22 °C leaf. **F:** Chloroplast after 7 days of 34 °C; the thylakoid arrangement is comparable to 32 °C, however, the plastoglobules are larger than control and 32 °C. **G:** Leaf section after 7 days of 36 °C; the tissue is less dense than in the 32 °C leaves, but still denser, with smaller cells than in control plants, and some cells exhibit a less turgid shape compared to lower temperatures. **H:** Chloroplast after 7 days of 36 °C; the thylakoids are arranged less regularly and are disturbed by the large plastoglobules. **I:** Leaf section after 3 days of 38 °C; the density of the tissue is comparable to the control leaf, but some cells also exhibit a less turgid shape. **J:** Chloroplast after 3 days of 38 °C; the chloroplast has a crescent shape, compared to the more lentil-like shape of chloroplasts of lower temperatures; the thylakoids are arranged similar to the 36 °C thylakoids; the size of the plastoglobules is comparable to 34 °C.

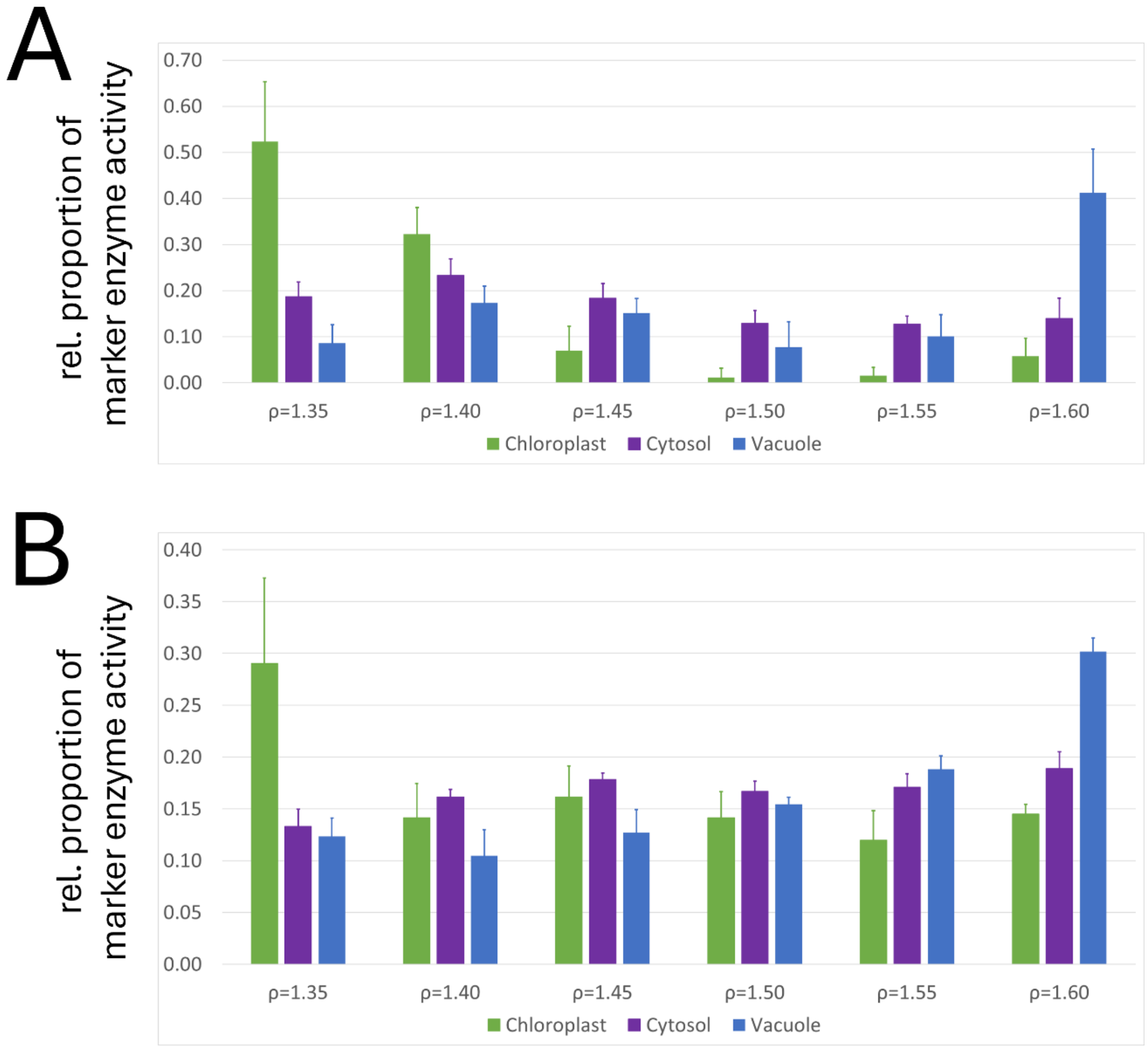

Supplementary Figure S4. Relative distribution of marker enzyme activities across non-aqueous density fractions. (**A**) control samples (22 °C), (**B**) heat acclimated samples (7 days at 34 °C). The relative proportion of enzyme activities in indicated on the ordinates (values 0-1), densities of fractions are indicated on the abscissa (ρ, [g cm^{-3}]). Green bars: alkaline pyrophosphatase (marker: plastids); purple bars: UDP-glucose pyrophosphorylase (marker: cytosol); blue bars: acidic phosphatase (marker: vacuole). Bars represent mean values, error bars represent standard deviation (n = 4).
