# Supplementary figures and images for: Subcellular plant carbohydrate metabolism under elevated temperature
Source: Plant Physiol. 2025 Apr 16;198(3):kiaf117. doi: 10.1093/plphys/kiaf117 (PMC12225672; doi:10.1093/plphys/kiaf117)

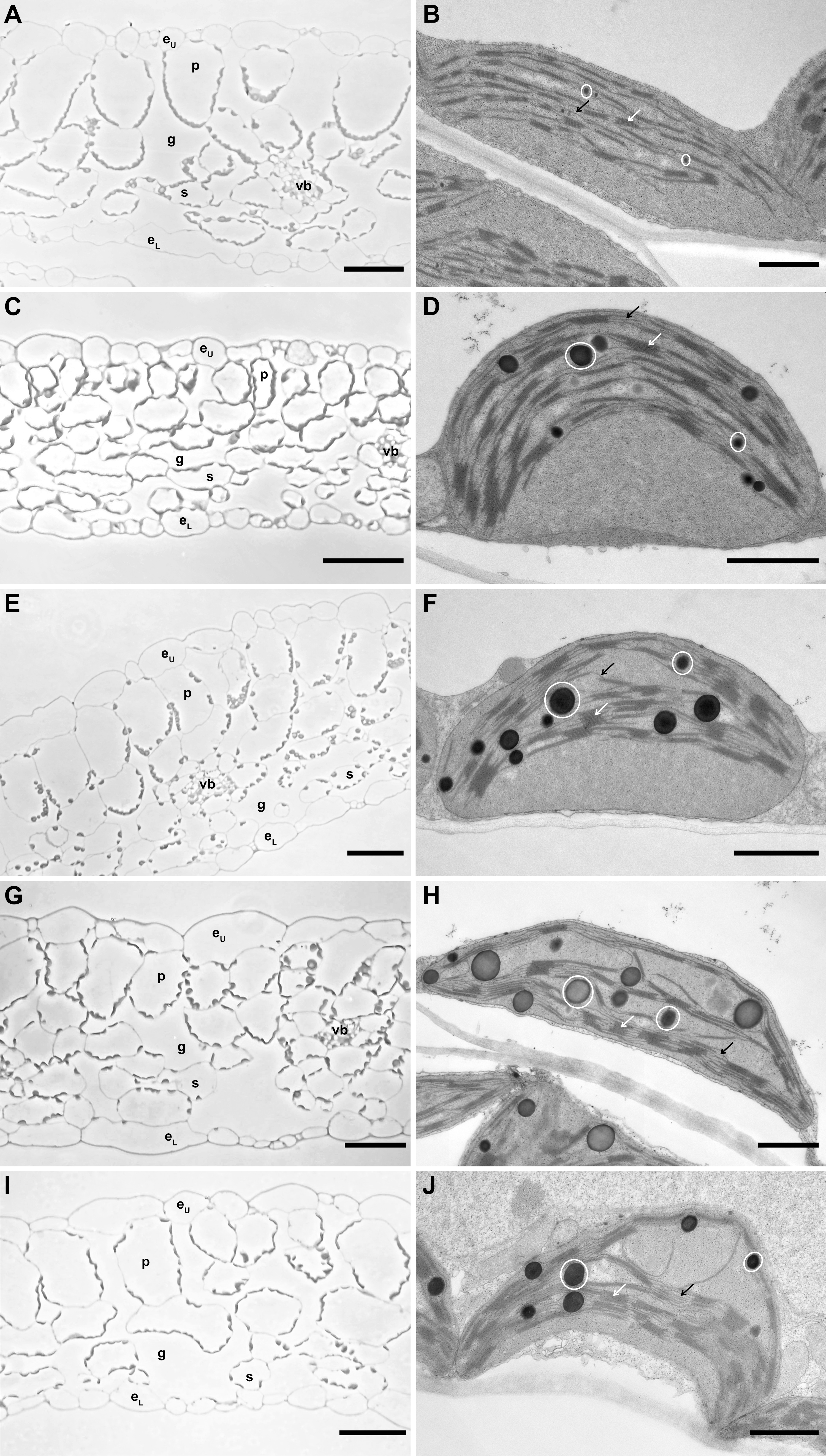

Supplement: kiaf117_Supplementary_Data [file kiaf117_supplementary_data.zip › FigureSF3_smaller.png]

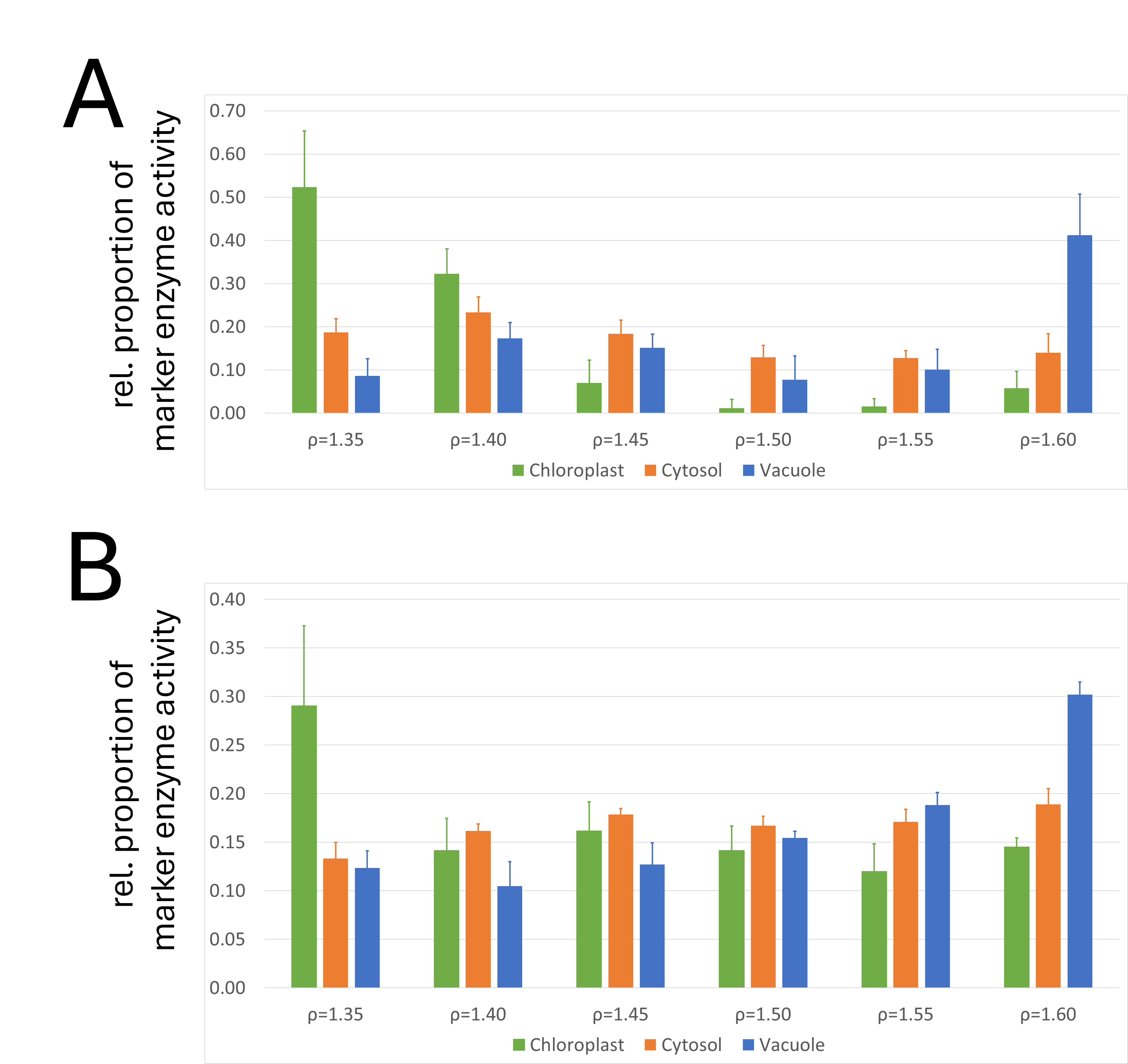

Supplement: kiaf117_Supplementary_Data [file kiaf117_supplementary_data.zip › Fig_SF4.tif]

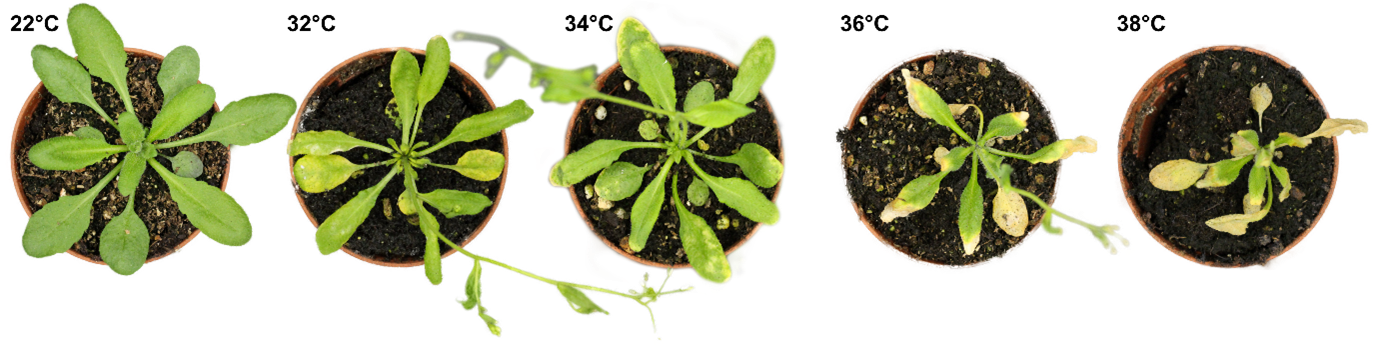

Supplement: kiaf117_Supplementary_Data [file kiaf117_supplementary_data.zip › SF1_phenotypes.tif]

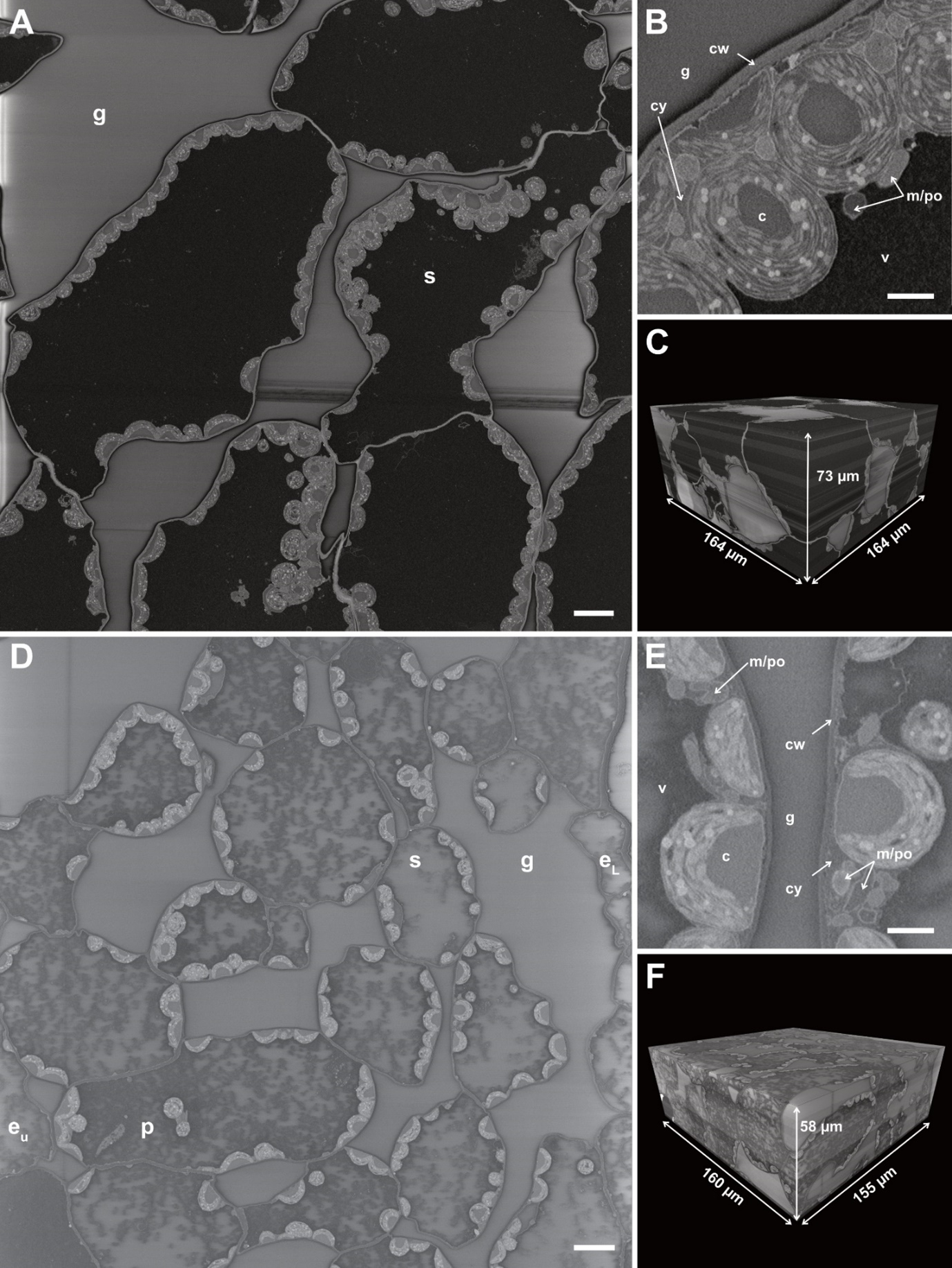

Supplement: kiaf117_Supplementary_Data [file kiaf117_supplementary_data.zip › SF2_SBFSEM.tif]
